# Prevalence, Subtype Distribution and Zoonotic Significance of *Blastocystis* sp. Isolates from Poultry, Cattle and Pets in Northern Egypt

**DOI:** 10.3390/microorganisms10112259

**Published:** 2022-11-14

**Authors:** Doaa Naguib, Nausicaa Gantois, Jeremy Desramaut, Nagah Arafat, Gaël Even, Gabriela Certad, Magali Chabé, Eric Viscogliosi

**Affiliations:** 1 CNRS, Inserm, CHU Lille, Institut Pasteur de Lille, U1019–UMR 9017–CIIL–Centre d’Infection et d’Immunité de Lille, University of Lille, F-59000 Lille, France; 2 Department of Hygiene and Zoonoses, Faculty of Veterinary Medicine, Mansoura University, Mansoura 35516, Egypt; 3 Department of Poultry Diseases, Faculty of Veterinary Medicine, Mansoura University, Mansoura 35516, Egypt; 4 GD Biotech-Gènes Diffusion, F-59000 Lille, France; 5 PEGASE-Biosciences (Plateforme d’Expertises Génomiques Appliquées aux Sciences Expérimentales), Institut Pasteur de Lille, F-59000 Lille, France; 6 Délégation à la Recherche Clinique et à l’Innovation, Groupement des Hôpitaux de l’Institut Catholique de Lille, F-59000 Lille, France

**Keywords:** *Blastocystis* sp., intestinal protozoa, poultry, cattle, pets, Africa, Egypt, molecular epidemiology, transmission, zoonosis

## Abstract

*Blastocystis* sp. is a widespread enteric protozoan that frequently infects human and animal groups. Despite its burden and zoonotic potential worldwide, epidemiological investigations remain limited in animal groups that come in contact with humans. Therefore, the largest survey ever conducted in North Africa was performed in Egypt with the aim to investigate the prevalence and subtype (ST) distribution of *Blastocystis* sp. in animals. For this purpose, a total of 889 fecal specimens were collected from chickens (217), cattle (373), dogs (144) and cats (155) from six governorates of northern Egypt. These specimens were then screened for the presence of *Blastocystis* sp. using a quantitative real-time PCR, followed by subtyping the isolates. The overall prevalence of *Blastocystis* sp. reached 9.2% (82/889), with the highest infection rates reported in chickens (17.0%) and domestic cattle (11.0%), highlighting an active circulation of the parasite in both animal groups. In contrast, the low prevalence in cats (2.6%) and the absence of the parasite in dogs suggested that pets are not natural hosts of *Blastocystis* sp. ST10 and ST14 were largely predominant in cattle, confirming that both STs represented cattle-adapted STs. The report of one ST3 and one ST4 isolate in this animal group could be explained by an accidental zoonosis from humans to animals. All but one of the subtyped isolates in poultry belonged to ST7, which was considered as an avian ST. The presence of a remaining isolate of ST14 likely reflected a transient infection from contact between birds and cattle feces. The same environmental contamination was also likely the source of the ST14 infection in three of the four positive cats, with the remaining animals infected by ST3 as the result of human-to-animal transmission. These occurrences and subtyping data, combined with those previously collected in the Egyptian population, implies that poultry could play a significant role as reservoir for zoonotic transmission, which would not be the case for cattle and pets.

## 1. Introduction

*Blastocystis* sp. is one of the most common intestinal protozoa that infects gastrointestinal tract of both humans and animals [1,2,3]. It has been recorded in human stool specimens from various geographical regions of the world, with prevalence rates ranging from 15 to 25% in European industrialized countries [4,5,6] and often exceeding 50% in developing countries, especially in Africa, due to poor sanitary conditions and the unavailability of effective water treatment [7,8,9]. *Blastocystis* sp. can spread to humans through either the consumption of fecal-contaminated water and food containing cyst formations of the protozoan or by intimate contact with infected individuals and animals. Thus, this protozoan could be considered anthroponotic or zoonotic in origin within the human population [10,11,12].

Even though the large majority of individuals infected by *Blastocystis* sp. do not present intestinal manifestations, recent in vitro and in vivo studies coupled with the identification of virulence factors have proven the pathogenicity of a proportion of isolates [13,14]. Consequently, a *Blastocystis* sp. infection is associated with non-specific gastrointestinal symptoms, including diarrhea, abdominal pain [10,15,16] and urticaria [17]. Interestingly, this protozoan has the potential to influence the gut microbiota compositions in humans and animals. Recent findings showed that a *Blastocystis* sp. infection is mostly associated with an increased bacterial richness in the human gut microbiome, leading to healthy gut microbiota [18,19,20]. In contrast, the presence of particular *Blastocystis* sp. isolates in a mouse model is accompanied by a decrease in beneficial bacteria, leading to an imbalance of the gut microbiota [21].

The comparison of the small-subunit rRNA (SSU rRNA) gene sequences was used to decipher the genetic diversity among *Blastocystis* sp. isolates of this parasite found in humans and animals. Based on nucleotide differences at this genetic locus, a total of 34 subtypes (ST1 to ST34) were reported in mammalian and avian hosts [22,23,24,25], with four (ST18-ST20 and ST22) considered to be invalid as they represent potential experimental artifacts [26]. Of the remaining 30 STs whose validity has not been questioned, 14 were found in the human population (ST1-ST10, ST12, ST14, ST16 and ST23) but with highly variable frequencies [27,28,29,30,31,32]. Nearly 90% of the human isolates subtyped so far belong to ST1 to ST4, showing marked variations between continents for ST4 and a general predominance worldwide for ST3 [27]. The remaining 10 STs are considered to be of animal origin and do not have a strong host specificity for infecting humans and various animal groups, as recently reviewed [1]. In particular, ST5 is predominantly found in pigs, ST6 and ST7 in birds, and ST10 and ST14 in cattle, which were also shown to infect humans, highlighting their potential for zoonotic transmission [9,29,32,33,34]. Hence, there is interest to conduct epidemiological surveys in animal groups having close and repetitive contact with humans.

A still-too-limited set of transmission studies, including animal samples, was carried out in different regions, such as Africa [1], although this region is considered to be at high risk of infection from this parasite. In North Africa, in particular in Egypt, only four surveys have been conducted to date, focusing on various animal groups, including poultry, bovid and pets [35,36,37,38]. However, these surveys included a restricted number of animal samples and used conventional methods of detection for *Blastocystis* sp., such as direct light microscopy and short-term xenic in vitro cultures, which are known to likely underestimate the prevalence of the parasite compared to the end-point or quantitative real-time PCR (qPCR) assays [39,40].

Therefore, the first purpose of the present study was to determine the prevalence and ST distribution of *Blastocystis* sp. isolates identified using qPCR and sequencing to screen of large cohorts of chickens, domestic cattle and pets (dogs and cats) in Egypt. In a second step, the molecular data collected allowed us to improve our understanding of the *Blastocystis* sp. epidemiology in these animal groups in North Africa and assess the potential risk of zoonotic transmission of this parasite.

## 2. Materials and Methods

### 2.1. Ethics Statement

All the fieldwork in this study was carried out in compliance with the Guide for the Care and Use of Laboratory Animals in Egypt and approved by the Research Ethical Committee of Faculty of Veterinary Medicine, Mansoura University with the code number R/99. The owner’s permission was obtained before the collection of fecal specimens.

### 2.2. Specimen Collection

**Figure 1:**
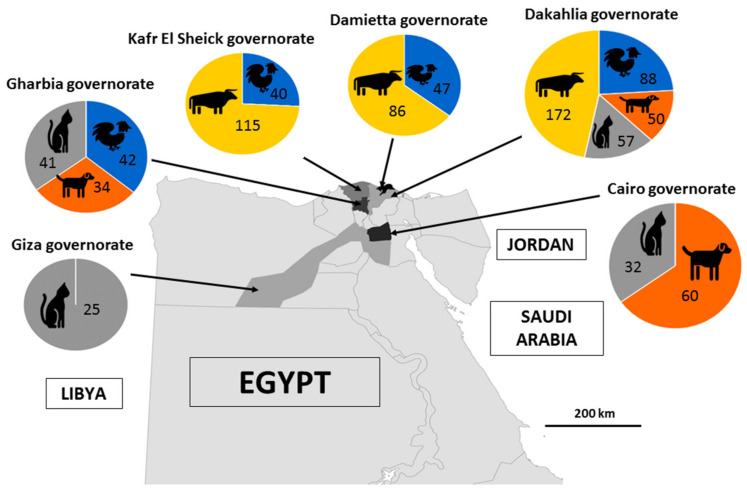
Study areas in Northern Egypt with the indication of the number of animals sampled for the selected species in each governorate.

A total of 889 fresh animal fecal specimens were randomly collected from cattle (*n* = 373), dogs (*n* = 144), cats (*n* = 155) and chickens (*n* = 217) in the governorates of Dakahlia, Gharbia, Damietta, Kafr El Sheikh, Cairo and Giza in Northern Egypt throughout 2021 (Figure 1). The standardized epidemiological data were recorded for each animal, such as age, sex, breed, location and lifestyle. The selected animals did not show any digestive symptoms and were, therefore, considered to be healthy. The dairy cattle sampled in this study were of the Holstein, Friesian or Baladi breed and were categorized into three groups according to their age (less than 3 months, between 3 and 6 months and more than 6 months). The chicken specimens were collected from farms such as broilers (*n* = 49), layers (*n* = 22) and breeders (*n* = 17) and from live bird markets, namely broilers (*n* = 129). Regarding cats and dogs, all the samples were obtained from shelters, veterinary clinics or pet shops. The cats were aged between 1 month and 4 years and were from the Shirazi, Chausie, African wild cat, Nile Valley Egyptian and Persian breeds. The dog samples were collected from German shepherd, Baladi, Rottweiler, Griffon and golden retriever breeds aged between 1 month and 5 years.

One specimen per study animal was collected using a sterile plastic cup either from the rectum of the animal using sterile gloves or immediately after defecation, except for chicken specimens. The fecal and cecum specimens from chickens were gathered from commercial farms and live bird markets. Five random droppings from various areas inside the farms were pooled and collected into one specimen. For the live bird market specimens, five ceca were collected from five randomly selected chickens belonging to the same batch aged from 38 to 116 days old, and the contents of the ceca were evacuated in a sterile plastic cup under complete aseptic condition, mixed and regarded as one specimen. Two grams from each of the fresh animal specimens were added to 2 mL of 2.5% potassium dichromate (*w*/*v* in water) (Sigma Life Sciences, Saint-Louis, MO, USA) in a sterile Falcon tube, thoroughly mixed and kept at 4 °C for conservation prior to the DNA extraction at the Pasteur Institute in Lille, France.

### 2.3. DNA Extraction

The stored fecal specimens were washed three times with distilled water using centrifugation at 3000× *g* for 10 min to remove the potassium dichromate prior to the DNA extraction. The supernatant was discarded, and the pellet was diluted with 1 mL of sterile water. The genomic DNA was extracted from 500 μL of the diluted pellet using the NucleoSpin 96 Soil kit (Macherey-Nagel GmbH & Co KG, Düren, Germany) following the recommended procedures from the manufacturer. The DNA was eluted in 100 μL of elution buffer and stored at −20 °C to await molecular analyses.

### 2.4. Blastocystis sp. Detection and Subtyping of Isolates

The specimens were examined for the presence of *Blastocystis* sp. by qPCR targeting the small subunit (SSU) rRNA gene using 2 μL of the extracted DNA and *Blastocystis*-specific primer pair BL18SPPF1/BL18SR2PP, as previously described [40]. The amplified fragment of approx. 300 bp length was sufficiently discriminating in terms of the sequence information for accurately subtyping the *Blastocystis* sp. isolates. Each PCR analysis was conducted in duplicate, using the *Blastocystis* sp. ST8 DNA obtained from an axenic culture as the positive control and reagent-grade water as the negative control. All the *Blastocystis* sp.-positive specimens were selected and the STs were identified using the sequence analysis of the purified qPCR products (Genoscreen, Lille, France; SANGER technology platform, 3730XL DNA Analyzer). For a significant proportion of the bovid specimens, a double trace was present during the analysis of the sequence chromatogram, suggesting a mixed infection of these specimens from two or more different *Blastocystis* sp. STs. Therefore, three of these specimens that presented mixed infections were randomly selected and re-analyzed by an end-point PCR using the same primer pair for qPCR. The PCR amplification was conducted in 50 μL containing a 5 μL 10× PCR buffer, 2 μL of MgCl2, 0.5 μL of dNTP (25 nM), 1.25 μL (10 nM) of each primer, 0.5 μL (5 U/μL) of HotStarTaq *Plus* DNA Polymerase (QIAGEN GmbH, Hilden, Germany) and 5 µL of Template DNA. The cycling protocol was started with an initial denaturation at 95 °C for 5 min, followed by 40 cycles of denaturation at 94 °C for 10 s, annealing at 55 °C for 15 s and extension at 72 °C for 40 s, with a final extension of 72 °C for 1 min. The PCR products were purified using the NucleoSpin Gel and PCR Clean-up kit (Macherey-Nagel GmbH & Co KG). The purified PCR products were then cloned in the T-vector TA pCR–TOPO 2.1 of the TOPO TA cloning kit (Invitrogen, Carlsbad CA, USA), following the recommendations from the manufacturer, and amplified in the One Shot TOP10 chemically competent *Escherichia coli* (Invitrogen). The minipreparations of plasmids were performed using the NucleoSpin Plasmid kit (Macherey-Nagel GmbH & Co KG). For each specimen, five positive clones exhibiting an insert of the expected size were randomly selected and sequenced on both strands. The obtained sequences in this study were deposited in GenBank under accession numbers OM827097-OM827168. The ChromasPro v2.6.6. software (www.technelysium.com.au/ChromasPro.html/, accessed on 22 January 2022) was used to assemble and edit the generated nucleotide sequences. The corresponding STs were identified by determining the exact match or closest similarity against all known *Blastocystis* sp. ST homologous sequences available in the National Centre for Biotechnology Information (NCBI), using the nucleotide basic local alignment search tool (BLASTn) program.

### 2.5. Statistical Analysis

For the statistical analysis, Fisher’s exact test was used to test the relationship between the different categorical variables (governorate, age, sex and breed for cattle; location, collecting area and production system for poultry). The multilevel logistic-mixed regression models were created to calculate the odds ratios (OR) and the 95% confidence interval (CI), considering the *Blastocystis* sp. prevalence and STs as the main outcomes. The *p*-value of 0.05 was selected as the limit for significance with a 95% confidence interval. The analyses were performed using the package statistics and odds ratio 2.0.1 from the R statistical computing program (Version 4.1.1 Date of release 10 August 2021; R Development Core Team, http://www.R-project.org, accessed on 10 June 2022).

## 3. Results and Discussion

To our knowledge, this study is the largest survey ever conducted on the molecular epidemiology of *Blastocystis* sp. in animals in North Africa, with the analysis of 889 samples collected from poultry, cattle, dogs and cats. These groups of animals are of particular interest to assess the zoonotic potential of this parasite, as they usually live in close and repeated contact with the human population. Of the 889 fecal samples collected from six governorates in Egypt and tested by qPCR for the presence of *Blastocystis* sp., 82 were positive for a significant average prevalence of 9.2%. Strikingly, the frequency of the parasite was extremely variable, ranging from 0% to 17.0%, depending on the group of animals (Table 1).

Among the 373 fecal samples collected from domestic cattle in three governorates (Dakahlia, Damietta and Kafr El Sheikh), 41 of them (11.0%) were shown to be positive for *Blastocystis* sp. (Table 1). In addition, the prevalence of *Blastocystis* sp. varied widely between the governorates. The risk of parasite infection in cattle was significantly lower in Damietta (4.7%) (OR: 0.330, CI: 0.097–0.853, *p* = 0.040) than in the Dakahlia (15.7%) and Kafr El Sheikh (8.7%) governorates, and reversely higher in the Dakahlia governorate (OR: 2.487, CI: 1.278–5.040, *p* = 0.009). Interestingly, the frequency of the parasite reported in the Kafr El Sheikh governorate (8.7%) was globally in the same range as previous observations in the same province (19.4%) that used direct light microscopy and an in vitro culture of fecal samples collected from a group of 190 cattle [37]. In contrast, the overall prevalence observed in the present study regardless of the governorate (11.0%) was much lower than the 72.2% previously reported in a cohort of 18 cattle samples from the Ismailia governorate, also located in northern Egypt, even if this animal population was too small to be significant [36]. In addition, a pooled frequency of 24.4% was calculated in a meta-analysis conducted worldwide and included a total of more than 9000 fecal samples [41]. However, it was similar to those observed using molecular diagnostic methods in cattle populations from different countries, such as China (9.5%) [42], Indonesia (9.4%) [43] and Turkey (11.3%) [44]. These variations in the prevalence of the parasite between countries, as between Egyptian governorates, can be explained largely by the different cattle housing and sanitary conditions in the farms, as already highlighted [1].

Regarding the sex of animals within the global cohort of cattle, the difference in the prevalence observed in this survey between males (19/201, 9.4%) and females (22/172, 12.8%) was not significant (OR: 0.712, CI: 0.368–1.367, *p* = 0.306). *Blastocystis* sp. was also identified in all of the tested breeds, with slightly significant higher infection rates in Baladi (14/180, 17.5%) than in Holstein (17/161, 10.5%) and Friesian (10/132, 10.6%) cattle (OR: 2.090, CI:1.015–4.152, *p* = 0.039). The age of the animals was also identified as a factor influencing the prevalence of *Blastocystis* sp. (Fisher exact test, *p* = 0.0005). More precisely, our data revealed that the parasite was significantly more frequently found in animals aged > 6 months (33/94, 35.1%; OR: 18.326, CI: 8.443–44.461, *p* = 3.8 × 10^–12^) and significantly less present in calves aged 3–6 months (4/99, 4.0%; OR: 0.270, CI: 0.079–0.696, *p* = 0.015) and <3 months (4/180, 2.2%; OR: 0.096, CI: 0.028–0.246, *p* = 1.29 × 10^–5^). These findings were concurred with the previous studies conducted in China [45] and Korea [46], which reported that calves aged 3 months or less, exhibited a significant lower occurrence of *Blastocystis* sp. than older groups of animals (3–11 months and >12 months). Similarly, in two surveys conducted in the United States, a low prevalence of 2.9% was observed in the former among a large cohort of over 2500 pre-weaned dairy calves of < 2 months of age [47]. The parasite was only identified in animals older than 3 months in the latter [48]. Suckling and weaning calves fewer than 6 months of age were fed and bred under better sanitary conditions than older calves raised outdoors. Therefore, these calves were less exposed to *Blastocystis* sp., which could likely explain these differences in parasite prevalence related to animal age groups.

Of the 41 cattle samples that tested positive from qPCR, 25 corresponded to single infections by either *Blastocystis* sp. ST3 (*n* = 1), ST4 (*n* = 1), ST10 (*n* = 13) or ST14 (*n* = 10) (Table 1 and Table 2). The remaining 16 positive samples presented mixed infections according to the resulting sequence chromatograms. This represented about 39% of the positive samples and confirmed the high incidence of mixed infections in domestic cattle as reported nearly worldwide [41]. According to our data, ST10 and ST14 were largely predominant, accounting for 92.0% of the isolates subtyped in our cattle cohort. In recent investigations gathering all available data regarding STs identified in domestic cattle around the world [1,41,49], approx. 16 STs have been reported to date with highly variable frequencies. Among these STs, ST10 and ST14 were found to be the most widely distributed in many countries, strongly suggesting that domestic cattle represent natural hosts for these two STs. Interestingly, these two STs were also identified in domestic cattle in a study conducted in the Egyptian governorate of Kafr El Sheikh, including only the subtyping of seven bovine isolates [37] (Table 2). In contrast, ST10 and ST14 were not reported in a second epidemiological survey performed in the Ismailia governorate, comprising 13 cattle isolates [36] (Table 2). However, in this latter study, the subtyping of isolates was performed by PCR using ST-specific primers that allowed for the detection of only ST1 to ST7. The non-typing of six isolates could, therefore, be representative of ST10 and/or ST14. In parallel, among the 16 mixed infections identified in our cattle samples, three were re-analyzed using end-point PCR, followed by cloning the PCR product and sequencing the positive clones in order to obtain a partial overview of the STs present in these specimens. Not surprisingly, these three samples showed mixed infections with ST10 and ST14, confirming the predominance and active circulation of these two STs in the Egyptian cattle cohort.

**Table 2:** ST distribution of *Blastocystis* sp. in domestic cattle in Egypt.

Number of Subtyped Isolates	Subtyping Method	*Blastocystis* sp. STs						MI ^a^	Not Typed	Reference
		ST1	ST3	ST4	ST5	ST10	ST14			
7	Sequencing	0	0	1	0	1	5	0	0	[37]
13	PCR-STS ^b^	2	3	0	2	0	0	0	6	[36]
41	Sequencing	0	1	1	0	13	10	16	0	Present study

^a^ MI, mixed infection. ^b^ STS, ST-specific sequence-tagged site.

Until only recently, ST10 and ST14 had not been found in the human population and, therefore, the risk of zoonotic transmission from bovids was considered to be minimal [49]. Nevertheless, the epidemiological surveys performed in the last two years in West Africa and Asia [9,29,32] revealed the presence of both STs in humans, possibly with a significant frequency as shown in Guinea (3.4% of the subtyped isolates). According to the latest review of all subtyping data available [32], these two STs have not been identified in the human population in Egypt. However, the cumulative molecular data showed a very large predominance of ST3 (323/478, 67.6% of the isolates), followed by ST1 (65/478, 13.6%), ST6 (41/478, 8.6%), ST7 (17/478, 3.5%), ST4 (19/478, 4.0%) and ST2 (13/478, 2.7%). Arguably, domestic cattle did not appear to represent a potentially relevant source of transmission of ST10 and ST14 parasites to humans in Egypt even if further large-scale studies have to be carried out to confirm this hypothesis.

The remaining two isolates that were subtyped in our cattle cohort belonged to ST3 and ST4. Interestingly, both STs were also reported to have low prevalence in the two previous studies conducted in the same animal group in Egypt (Table 2). As recently reviewed [1], ST3 was commonly found in domestic cattle worldwide while ST4 has so far been identified in the same animal group only in cohorts from China [45] and the USA [47]. ST3 was shown to be largely predominant in the human population [27] as it is also the case in Egypt (67.6% of the subtyped isolate) [32]. In addition, the prevalence of ST4 in humans varies from one continent to another, notably rare in Africa even though it was identified in 4% of the Egyptian subtyped isolates. ST3 and ST4 are thus considered to be linked to human infection [11,12] and not adapted to bovids. Consequently, the infection by both STs in domestic cattle could likely be explained by an accidental contamination from humans to animals, as previously suggested for a Lebanese cohort of livestock [49].

The higher prevalence of *Blastocystis* sp. was observed in the cohort of poultry (37 positive samples on 217, 17.0%). However, this frequency was much lower than the previously reported infection rates based on the in vitro cultivation detection method, reaching 82.5% in a group of 57 chickens sampled in the Ismailia governorate in Egypt [36], and 30.5% from a light microscopy examination of 200 intestinal samples of chickens collected in the Assiut governorate located in central Egypt [38]. Such variations in the prevalence between these surveys can be explained by the different geographical locations and lifestyles of the animals that, in some cases, may facilitate the circulation of the parasite. The study conducted in the Ismailia governorate included free-range chickens reared in rural areas where the risk of a *Blastocystis* sp. infection was high due to contaminated environmental sources, in particular drinking water spots. Globally, large differences in the parasite frequency determined by PCR were reported within chicken populations, ranging from 4.0% in Australia to 34.2% in Indonesia, with an average of 25% [1].

By analyzing the present data separately for each governorate, the mean prevalence of the parasite in poultry ranged from 11.4% (Dakahlia governorate) to 23.8% (Gharbia governorate). However, no significant difference in frequency was found to be associated with the geographical area (Fisher’s exact test, *p* = 0.165). In addition, the difference in the prevalence observed in poultry collected from live bird markets (25/129, 19.4%) and from farms (12/88, 13.6%) was not significant (Fisher’s exact test, *p* = 0.358). In our study, the poultry production system also did not represent a risk for parasite infection since the differences in the prevalence reported in broilers (33/178, 18.5%), layers (3/22, 13.6%) and breeders (1/17, 5.9%) were not significantly different (Fisher’s exact test, *p* = 0.452), even though the number of chickens was quite low for two of these categories.

In the context of our study, the 37 *Blastocystis* sp. isolates from poultry were subtyped. Interestingly, 36 of them belonged to ST7 (97.3%), while the last isolate was identified as ST14. The presence of only ST7 was also reported in small samplings of domestic chickens from Indonesia [50] and the Ivory Coast [51]. In the governorate of Ismailia in Egypt, ST7 was also described as predominant (73.5% of the subtyped isolates), followed by ST6 [36]. These ST distributions confirmed the recent previous molecular surveys [1], supporting that birds represent natural hosts for ST7, which is considered to be one of the “avian STs” together with ST6. Additionally, as previously discussed, more than 12% of the human isolates characterized in the Egyptian population belonged to ST6 and ST7, highlighting the high zoonotic potential of avian STs in this country. The zoonotic transmission of ST6 was, for instance, clearly demonstrated in Lebanon from contact between poultry and workers in slaughterhouses, likely due to the exposure to animal feces [34]. The last isolate identified in the present study in poultry belonged to ST14, which is considered as a bovine-adapted ST, as described above. Therefore, the poultry infection by *Blastocystis* sp. ST14 is likely reflected as a transient contamination through the contact between birds and cattle feces.

A large number of fecal samples from pets were also analyzed in the present study. The overall prevalence observed for the parasite in feline specimens was extremely low, reaching only 2.6% (4/155). Similarly, *Blastocystis* sp. was absent in eight cat fecal samples collected in the governorate of Ismailia and screened using short-term xenic in vitro cultures [36]. Globally, molecular data still remain scarce for cats, as recently summarized, since only a few epidemiological surveys provided both prevalence and subtyping data [1,52]. Currently, only five of these studies included a significant number of samples (more than 100) and showed a prevalence of 0.6% in China [53] and South Korea [54], 3.6% in Turkey [55], 11.7% in the USA [56] and 17.7% in Iran [57]. In parallel, *Blastocystis* sp. was not detected in feline samples in surveys conducted in Spain [58] or Poland [59]. In the present study, the four cats infected with *Blastocystis* sp. were from various breeds sampled in veterinary clinics, shelters or pet shops, with an age range between 2 to 18 months (Table 3). The prevalence of the parasite was too low to statistically test for a possible relationship with age, species or the welfare of animals.

Interestingly, three of the cats were colonized by ST14 isolates while the remaining cat was colonized by an ST3 isolate. This distribution of the STs was very surprising since ST14 is considered to be bovine-adapted ST, as previously discussed. Furthermore, to our knowledge, only one other case of an ST14 infection in cats was described in a recent study conducted in Iran [55]. The presence of this ST in cats was, therefore, likely related to an exposure to cattle feces, as suggested above for one chicken specimen. Concerning ST3, it was more commonly observed in cats, although molecular data still remain very limited [1,52]. However, given the high prevalence of ST3 in the Egyptian human population and the close contact between cat owners and animals, the infection of ST3 in cats is most likely the result of human-to-animal transmission.

*Blastocystis* sp. was not detected in any of the 144 canine stool samples tested in our study. Similarly, 21 dog fecal samples collected in the Ismailia governorate were also shown to be negative by culture method [36]. The occurrence of the parasite was reported to be about only 3.0% using direct -light microscopy in a population of 130 domestic dogs from the Sharkia and Qalyubia governorates, also located in northern Egypt [35]. Using molecular methods, the parasite was not identified in dogs in numerous countries, including Spain [58], Poland [59] and Greece [60], but was found with low occurrence in France (3.4%) [61], Australia (2.5%) and Cambodia (1.3%) [62]. In a survey conducted in Southern China [63], the authors showed significant differences in the prevalence of *Blastocystis* sp. depending on the care conditions of the animals. While the prevalence of the parasite was 5.4% in shelter dogs, it was null in other surveyed canine populations, including household, breeding and pet market dogs. This could be explained by the fact that, before entering the shelters, the dogs roamed freely and were, therefore, more exposed to humans and animal feces and, consequently, to parasite infections. Accordingly, a prevalence of *Blastocystis* sp. exceeding 20% was reported in dogs housed in Italian rescue shelters [64] and a higher occurrence of the parasite was observed in cohorts of stray dogs in India [62]. However, although 62 of the 144 samples tested in our study were from shelter dogs, the parasite was not found in the corresponding fecal samples. This likely means that the present cohort of dogs living in shelters, households and pet shops in northern Egypt were kept in good sanitary conditions with limited contact with the outside, properly fed and regularly examined by veterinarians, protecting them from infection by *Blastocystis* sp.

Due to the low prevalence of the parasite in both dogs and cats in our survey, the diversity of the STs identified in the literature in these two groups and the absence of any predominant ST for either of them, these pets would not represent natural hosts of *Blastocystis* sp., but rather act as occasional carriers for this parasite. Therefore, pet cats and dogs pose a minimal zoonotic risk of *Blastocystis* sp. infection to their human companions at least in Northern Egypt.

## 4. Conclusions

The present large-scale survey expands our knowledge on the role of animals in the molecular epidemiology of *Blastocystis* sp. in North Africa, more specifically in Egypt. Overall, our results highlight the common infection of domestic cattle and poultry by this parasite and confirm that ST10 and ST14 are bovine-adapted STs, while ST7 is avian-adapted. In contrast, the occurrence and circulation of *Blastocystis* sp. is extremely limited in dog and cat populations, according to the low prevalence observed in these animal groups, strongly suggesting that pets do not represent natural hosts of the parasite. By comparing the overall ST distribution between domestic cattle or poultry and humans in Egypt, it appears that poultry, but not cattle, could play a significant role as a reservoir for zoonotic transmission, hence the interest in developing protective measures to avoid this spread. Despite intimate contact with humans, it is unlikely that pets represent a significant source of zoonotic transmission to owners and animal handlers. The data also encourage us to conduct further investigations in Egypt regarding other animal hosts of *Blastocystis* sp. that are in contact with human populations, such as goats, camels, sheep and rabbits, to complete the epidemiology of this parasite. Additionally, to better understand the transmission dynamics of *Blastocystis* sp., One Health approaches have to be developed by screening human, animal and environmental samples collected within the same restricted geographic area.

## Figures and Tables

**Table 1:** Prevalence and ST distribution of *Blastocystis* sp. in various animal groups sampled in different Egyptian governorates.

Animal Group	Governorate	No. Examined	No. Positive (%)	STs (No.)
Cattle	Dakahlia	172	27 (15.7)	ST10 (9); ST14 (7); MI ^a^ (11)
	Gharbia	0	-	-
	Damietta	86	4 (4.7)	ST10 (2); ST14 (1); MI ^a^ (1)
	Kafr El Sheikh	115	10 (8.7)	ST3 (1); ST4 (1); ST10 (2); ST14 (2); MI ^a^ (4)
	Cairo	0	0	-
	Giza	0	-	-
**Subtotal**		**373**	**41 (11.0)**	**ST3 (1); ST4 (1); ST10 (13); ST14 (10); MI ^b^ (16)**
Chickens	Dakahlia	88	10 (11.4)	ST7 (10)
	Gharbia	42	10 (23.8)	ST7 (10)
	Damietta	47	11 (23.4)	ST7 (10); ST14 (1)
	Kafr El Sheikh	40	6 (15.0)	ST7 (6)
	Cairo	0	-	-
	Giza	0	-	-
**Subtotal**		**217**	**37 (17.0)**	**ST7 (36); ST14 (1)**
Cats	Dakahlia	57	1 (1.7)	ST14 (1)
	Gharbia	41	1 (2.4)	ST14 (1)
	Damietta	0	-	-
	Kafr El Sheikh	0	-	-
	Cairo	32	0 (0)	
	Giza	25	2 (8.0)	ST3 (1); ST14 (1)
**Subtotal**		**155**	**4 (2.6)**	**ST3 (1); ST14 (3)**
Dogs	Dakahlia	50	0 (0)	-
	Gharbia	34	0 (0)	-
	Damietta	0	-	-
	Kafr El Sheikh	0	-	-
	Cairo	60	0 (0)	-
	Giza	0	-	-
**Subtotal**		**144**	**0 (0)**	**-**
**Total**		**889**	**82 (9.2)**	**ST3 (2); ST4 (1); ST7 (36); ST10 (13); ST14 (14); MI (16)**

^a^ MI, mixed infection. ^b^ Mixed infections by ST10 and ST14 in three selected samples. The 13 remaining samples corresponded to mixed infections with unidentified STs.

**Table 3:** Data on infected cats with *Blastocystis* sp. in Egypt.

Governorate	Sex	Age in Months	Life Style	Breed	ST
Gharbia	F	18	Veterinary clinic	Persian	ST14
Dakahlia	F	2	Shelter	Nile Valley Egyptian	ST14
Giza	M	9	Pet shop	Persian	ST3
Giza	F	12	Veterinary clinic	Shirazi	ST14

## Data Availability

Not applicable.
